# Seeing eye to eye: a modified Delphi method-based multidisciplinary expert consensus on the diagnosis and treatment of vernal keratoconjunctivitis

**DOI:** 10.1007/s00431-024-05776-0

**Published:** 2024-09-26

**Authors:** Daniele Giovanni Ghiglioni, Gaia Bruschi, Elena Chiappini, Alessandra Consales, Pia Allegri, Pasquale Aragona, Stefano Bonini, Roberto Caputo, Fabio Cardinale, Massimo Landi, Andrea Leonardi, Gian Luigi Marseglia, Francesca Mori, Marcella Nebbioso, Paolo Nucci, Silvia Osnaghi, Ugo Procoli, Edoardo Villani, Anna Maria Zicari, Michele Miraglia Del Giudice

**Affiliations:** 1 Fondazione IRCCS Ca’ Granda Ospedale Maggiore Policlinico, SC Pediatria Pneumoinfettivologia, Milan, Italy; 2 Department of Clinical Sciences and Community Health, Dipartimento Di Eccellenza 2023-2027, University of Milan, Milan, Italy; 3 Department of Health Sciences, University of Florence, Florence, Italy; 4 Pediatric Infectious Diseases Unit, Meyer Children’s Hospital IRCCS, Florence, Italy; 5 Ocular Inflammatory Diseases Referral Center Head, Rapallo Hospital, Genova, Italy; 6 Department of Biomedical Sciences, University of Messina, Messina, Italy; 7 Ophthalmology Complex Operative Unit, University Campus Bio-Medico, Rome, Italy; 8 Pediatric Ophthalmology Unit, Meyer Children’s Hospital IRCCS, Florence, Italy; 9 Pediatric Department, Pediatric Hospital Giovanni XXIII, Azienda Ospedaliero-Universitaria Policlinic of Bari, Bari, Italy; 10 Department of Medical Sciences-Graduate School of Allergology and Clinical Immunology, University of Turin, Turin, Italy; 11 Department of Neuroscience, Ophthalmology Unit, University of Padua, Padua, Italy; 12 Pediatric Unit, Department of Clinical, Surgical, Diagnostic and Pediatric Sciences, University of Pavia, Pavia, Italy; 13 Pediatric Clinic, Fondazione IRCCS Policlinico San Matteo, Pavia, Italy; 14 Allergy Unit, Meyer Children’s Hospital IRCCS, Florence, Italy; 15 Department of Sense Organs, Sapienza University of Rome, Rome, Italy; 16 Department of Biomedical, Surgical and Dental Sciences, University of Milan, Milan, Italy; 17 Eye Clinic, IRCCS MultiMedica, Milan, Italy; 18 Ophthalmology Complex Operative Unit, Children’s Hospital Giovanni XXIII Azienda Ospedaliero-Universitaria “Consorziale Policlinico”, Bari, Italy; 19 Department of Clinical Sciences and Community Health, University of Milan, Milan, Italy; 20 Department of Maternal Infantile and Urological Science, Sapienza University of Rome, Rome, Italy; 21 Department of Woman and Child and General and Specialized Surgery, University of Campania Luigi Vanvitelli, Naples, Italy

**Keywords:** Vernal keratoconjunctivitis, VKC, Ocular allergy, Consensus

## Abstract

Vernal keratoconjunctivitis (VKC) is a chronic, recurrent, inflammatory disease that affects both eyes, often with asymmetric severity, potentially causing major visual complications. The seasonal management of VKC can be challenging, especially when specialists with different diagnostic and therapeutic approaches need to be consulted. The aim of this expert panel was to reach a national consensus among pediatric allergologists and ophthalmologists on the diagnosis and treatment of VKC. This consensus was developed by an expert panel of 17 Italian pediatric allergologists and ophthalmologists with over a decade of experience. Ten statements on VKC diagnosis and treatment formulated after a thorough review of current literature were evaluated by the panelists. The level of agreement was quantitatively assessed using a 5-point Likert scale. Consensus was reached if ≥ 75.0% of panelists agreed to any given statement. The consensus emphasizes the importance of evaluation by multispecialty reference centers or experienced specialists for accurate diagnosis. Prompt diagnosis, especially during active phases, is crucial and should occur before corticosteroid therapy. The Bonini score from 2007 is the preferred tool for VKC assessment, although future revisions may be considered. Short cycles of topical corticosteroids should be preferred over prolonged use, even during immunomodulatory therapy. When cyclosporine fails, tacrolimus should be considered. *Conclusion*: This is the first consensus on the management of VKC that has gathered the expert opinions of both pediatricians and ophthalmologists. The outcome of this multidisciplinary effort provides a uniform approach to VKC diagnosis and treatment, thereby facilitating patient management across the country.

**Table:** 

**What is Known:** • *Vernal keratoconjunctivitis (VKC) is a chronic recurrent ocular disease particularly prevalent in the pediatric population.* • *Despite its relevance, there is a lack of standardized approaches shared between pediatricians and ophthalmologists, leading to notable variations in clinical practice.*
**What is New:** • *This expert panel, comprising 17 pediatric allergologists and ophthalmologists, has reached a national consensus to provide standardized guidance for VKC management.* • *The consensus emphasizes the importance of a multidisciplinary approach to managing VKC, ensuring consistent and effective patient care.*

**Supplementary Information:**

The online version contains supplementary material available at 10.1007/s00431-024-05776-0.

## Introduction

Vernal keratoconjunctivitis (VKC) is a rare chronic inflammatory ocular disease with seasonal exacerbations that can potentially impact vision (Table 1). Its prevalence is estimated to range from 0.7 to 3.3 cases per 10,000 individuals, with 0.3 to 1.4 cases per 10,000 classified as severe [1–4]. VKC typically manifests between the ages of 3 and 10 and is more common in pre-pubertal males. The pathogenesis of VKC is still incompletely understood: allergological, immunological, endocrinological, genetic, and environmental factors appear to be involved, although the precise contribution of each remains unclear [5–7]. Nevertheless, VKC is currently classified as an allergic disease [8–11].

**Table 1:** Main clinical characteristics of VKC

Chronic relapsing course with flare-ups in the spring–summer period and improvement or disappearance of symptoms in the winter period, except in the most severe and persistent forms
Extreme clinical variability based on environmental, genetic, hormonal, immunological, and allergological factors (the pathogenetic role of each factor has yet to be defined)
Unpredictable evolution of the disease, which tends to persist for an extended period of time, frequently into adolescence
Current absence of biochemical markers that correlate with the evolution of the disease and can be easily used in daily practice
Resolution during or after puberty, although there are a few cases of persistence or reactivation into adulthood

The classic symptoms of VKC are itching, photophobia, tearing, slimy-stringy mucus secretions, burning, and pain with blurred vision [5]. Notable clinical signs include conjunctival hyperemia, giant cobblestone-like papillae on the superior tarsal conjunctiva, limbal infiltrate, Horner-Trantas dots, palpebral ptosis, or pseudoptosis [5]. According to the site affected by the inflammation, VKC can be classified into three forms: limbal, tarsal, and mixed [5, 12]. Chronic inflammation and the associated tissue remodeling may lead to long-term conjunctival complications and severe visual impairments, especially in cases of corneal involvement [5, 12, 13]. The diagnosis of VKC can take several months, and during this period, treatment may be suboptimal [14].

Globally, VKC is primarily managed by eye specialists, as evidenced by the fact that most international recommendations and guidelines have been authored by ophthalmologists [12, 15–18]. However, VKC’s classification as an allergic disease and its predominant occurrence in pediatric patients highlight the essential role of pediatric allergologists in its management.

In Italy, limited national collaboration between pediatric allergologists and ophthalmologists has so far resulted in a variety of therapeutic approaches nationwide, favored by the diverse clinical manifestations in different local contexts [1, 3, 7, 12, 15–17, 19, 20]. Diagnostic delays, suboptimal treatment, and lack of standardized guidelines contribute to patient and family disorientation and increased risk of long-term sequelae [14, 21].

The aim of this expert panel was to reach a national consensus to provide both general practitioners and specialists with clear guidance on the diagnosis and treatment of VKC. Including both pediatric allergologists and ophthalmologists in this multidisciplinary panel aimed to leverage their respective expertise to develop comprehensive management strategies. Furthermore, fostering consistency among the two medical specialties primarily involved in VKC management aimed to reduce confusion among patients and parents, potentially originating from varying information and recommendations provided by different healthcare professionals.

## Materials and methods

We adhered to the ACCORD (ACcurate COnsensus Reporting Document) guidelines for reporting the methodology and results of the present consensus [22].

A modified Delphi method was employed to establish consensus among a panel of expert clinicians and researchers on the optimal diagnosis and treatment of VKC. The modified Delphi method was chosen to ensure anonymity, accommodate the geographical dispersal of experts, and provide a structured approach to systematically gather and integrate collective input.

The expert panel was assembled based on the recognized expertise of the panelists in the fields of pediatric allergology and ophthalmology. The expert panel comprised 17 experts affiliated with Italian ophthalmological and pediatric university departments and hospitals. The panelists were selected by SIAIP (Italian Society of Pediatric Allergy and Immunology) and SIOPS (Italian Society of Pediatric Ophthalmology and Strabismus), including authors of high-quality literature relevant to the subject area, to incorporate a broad spectrum of expertise. The expert panel included 10 pediatric ophthalmologists specialized in anterior chamber diseases and 7 pediatric allergologists, all with at least 10 years of experience in the management of VKC within their specific fields. The choice to include 17 panelists was made to ensure an odd number for conclusive decision-making, while balancing the diverse expertise needed from both pediatric ophthalmologists and allergologists and maintaining a manageable size for efficient discussion. Recruitment was through email invitations by the coordinator (D.G.G.). All invited panelists accepted to participate to the expert panel. Before starting the consensus exercise, all panelists were asked to disclose any conflicts of interest. The coordinator (D.G.G.), reworker (G.B.), and research methodology consultant (E.C.) determined that the lack of conflicts of interest among the majority of panelists adequately mitigated the potential risk of bias.

The consensus development process involved a series of structured iterative rounds of communication. A preliminary face-to-face meeting was held to define the scope of the consensus and the issues that needed to be addressed.

A literature search was conducted by the coordinator (D.G.G.) and the reworker (G.B.), who searched PubMed database for articles published up to September 2022. No specific year was chosen for the oldest literature. The search was not restricted to English-only papers. Search terms used included vernal, vernal keratoconjunctivitis, and VKC. The level of evidence was evaluated and agreed upon by the coordinator (D.G.G.) and the reworker (G.B.), with only high-quality evidence (i.e., controlled studies, large uncontrolled studies, comprehensive narrative reviews, systematic reviews and metanalyses) being taken into consideration.

Key statements were then formulated based on the preliminary literature search and expert knowledge through collaboration between the coordinator (D.G.G.), the reworker (G.B.), and the research methodology consultant proposed by SIAIP and approved by SIOPS (E.C.). These statements covered essential aspects of diagnosis (5 statements) and treatment (5 statements) of VKC. The existing scientific evidence gathered from the preliminary systematic review and supporting each key statement was summarized and presented to the panelists as part of the voting process (Suppl. Table 1).

Validation of key statements occurred through a formal anonymous survey conducted on an online platform organized by the coordinator (D.G.G.), the reworker (G.B.), and the research methodology consultant (E.C.). Panel members were asked to anonymously rate their agreement with the 10 key statements on a 5-point Likert scale. In each case, members could only select one option: “Strongly agree,” “Agree,” “Neutral,” “Disagree,” and “Strongly disagree.” The reworker (G.B.) and the research methodology consultant (E.C.) did not take part in the voting process. The responses were collected by the research methodology consultant (E.C.) and kept blinded from the coordinator (D.G.G.) and the reworker (G.B.) to ensure anonymity and impartial evaluation.

Consensus was predefined by the coordinator (D.G.G.), reworker (G.B.), and research methodology consultant (E.C.) as reaching at least 75.0% agreement (agree or strongly agree) among panel members for each statement, as is common practice [23]. In cases of disagreement, revisions to the key statements were made based on collective feedback, followed by up to three rounds of anonymous revoting. This iterative process ensured thorough reevaluation and refinement of the statements based on collective input. Anonymity was maintained throughout all stages to prevent biases.

The final statements were derived from aggregated expert panel responses, integrating quantitative ratings and qualitative insight. All authors approved the final statements.

## Results

The present consensus exercise was conducted from September 2022 to June 2023. Each round of voting took 1 month, followed by a 2-month period dedicated to evaluating responses and refining the statements accordingly.

The panelists expressed their degree of agreement to a set of 10 statements on the diagnosis and treatment of VKC (Suppl. Table 1). The 10 final statements are reported in Table 2.

**Table 2:** The 10 final statements on diagnosis (5 statements) and treatment (5 statements) of VKC, as discussed and agreed upon by the expert panel members

**Diagnosis**	
**Statement #1**	For the diagnosis and follow-up of VKC, it is recommended to conduct ophthalmological and allergological examinations. In severe forms, seeking advice from other expert medical specialists, possibly in multispecialty reference centers, is advisable
**Statement #2**	Multispecialty reference centers should develop written instructions and information for patients, their families, and primary care physicians/pediatricians regarding the in-home management of mild forms of VKC
**Statement #3**	The initial clinical diagnosis and assessment of VKC exacerbations should be promptly conducted in patients exhibiting active disease who have not received topical or systemic corticosteroid therapy for a minimum of 7 days
**Statement #4**	The severity of the disease should be assessed through a score shared by reference centers including signs, symptoms, and the presence of aggravating factors
**Statement #5**	Conjunctival cytology currently plays a limited diagnostic role in everyday clinical practice and is often reserved to clinical trials. Its use is further constrained by the necessity for skilled personnel, aiming to minimize both the time and costs of implementation, as well as potential stress for the patient. In rare instances where the clinical differential diagnosis poses particular challenges, conjunctival cytology may offer supplementary insights contributing to the overall diagnostic process
**Treatment**	
**Statement #6**	Supportive measures (particularly sunglasses, visor cap, artificial tears) and antihistamines and topical mast cell stabilizers in case of hitching and/or known allergy should be recommended in the in-home management of all forms of VKC. In milder forms, the proper use of these aids can effectively control the disease, while in moderate forms it can delay the need for corticosteroids and immunomodulators
**Statement #7**	In individuals with VKC, topical steroid therapy should be prescribed and monitored by the expert ophthalmologist and/or the multispecialty reference center. The general physician/pediatrician should be informed of the therapy to facilitate its correct administration and schedule ophthalmological visits based on clinical evolution and according to the indications of the multispecialty reference center
**Statement #8**	In mild forms with periods of recrudescence of less than 3 months, steroids should not be used or should be used in short cycles of 3–5 days 1–2 times a month. In moderate forms and with periods of recrudescence of 3 or more months, steroids should be used in short cycles of 3–5 days. If 3 or more steroid cycles are needed in a month to control the disease, they should be replaced with immunomodulators. In severe forms requiring treatment with immunomodulators, steroids should be promptly used as supportive anti-inflammatory therapy for a brief duration at first, followed by short and repeatable 3–5 day cycles as rescue therapy, if needed
**Statement #9**	In severe cases of VKC, higher potency steroids are preferable to lower potency steroids. Steroid eye drops with lower ocular penetration and fewer eye-irritating preservatives/excipients should also be preferred
**Statement #10**	Tacrolimus 0.1% galenic eye drops are an alternative in case of ineffective therapy with cyclosporine eye drops

Fifteen (15) out of 17 experts (88%) agreed on the importance of reference centers for the diagnosis and management of severe forms of VKC. The panelists agreed that, in addition to pediatric allergologists and ophthalmologists, other specialists, such as immunologists and dermatologists, may play a supportive role and offer valuable insights for a more comprehensive management of patients with VKC within multispecialty reference centers.

Fifteen (15) out of 17 experts (88%) were in favor of the development of written instructions and information for the patient, the family, and the general physician/pediatrician by multispecialty reference centers. The panelists agreed that a shared diagnostic-therapeutic protocol discussed by experts provides clear indications and promotes knowledge among general healthcare professionals (i.e., those without specific expertise in VKC).

Eighty-two percent (82%) of the panelists agreed that the first diagnosis and the diagnosis of subsequent VKC exacerbations must be promptly made in patients with active disease not currently on corticosteroid therapy and who have not received it for at least the previous week.

Ninety-four percent (94%) of the panel agreed on the importance of a diagnostic score shared among reference centers. This score could assist reference centers in developing protocols applicable by general healthcare professionals.

Most panelists (94%) agreed that the conjunctival cytological examination should be reserved for research purposes and, in clinical practice, limited to cases of VKC with unclear clinical findings.

Regarding therapy, 88% of the panel members agreed that supportive measures should be recommended in all forms of VKC.

Eighty-two percent (82%) of the panelists agreed that the general physician/pediatrician should be familiar with topic corticosteroid therapy, albeit prescribed and monitored by expert ophthalmologists and/or multispecialty reference centers.

The majority of the panelists (88%) agreed on the use of steroids for different severity levels of VKC. The panelists emphasized the limited use of steroids in mild forms with short recrudescence periods and highlighted the lack of scientific evidence to support the practice of employing steroids in decreasing cycles of 15–20 days for mild forms, as observed in certain local contexts.

Eighty-two percent (82%) of the experts agreed that, in cases of severe VKC, higher potency steroids with lower penetration and fewer irritating preservatives/excipients and cationic surfactants (benzalkonium chlorides, cetalkonium chloride, sodium hydroxide, hydrochloric acid, alcoholic solutions) are preferable. In severe forms, such corticosteroids are most effective when used as rescue therapy during treatment with immunomodulators.

Finally, 82% of the experts concurred that, when cyclosporine fails to control VKC, as reported in 8–15% of cases in the literature [19], 0.1% tacrolimus galenic eye drops should be employed.

## Discussion

VKC is a chronic keratoconjunctivitis characterized by a seasonal relapsing clinical course [5, 12]. Although it is usually classified as an allergic conjunctivitis, its pathogenesis is still unclear. In Italy, VKC has historically been managed through ad hoc and seasonal interventions by various professionals, not all of whom have a specific background and expertise in VKC. This has resulted in a lack of a unified diagnostic and therapeutic strategy. Consequently, establishing a national consensus on diagnostic pathways and therapeutic protocols was crucial to ensure consistent VKC management across the country.

**Fig. 1:**
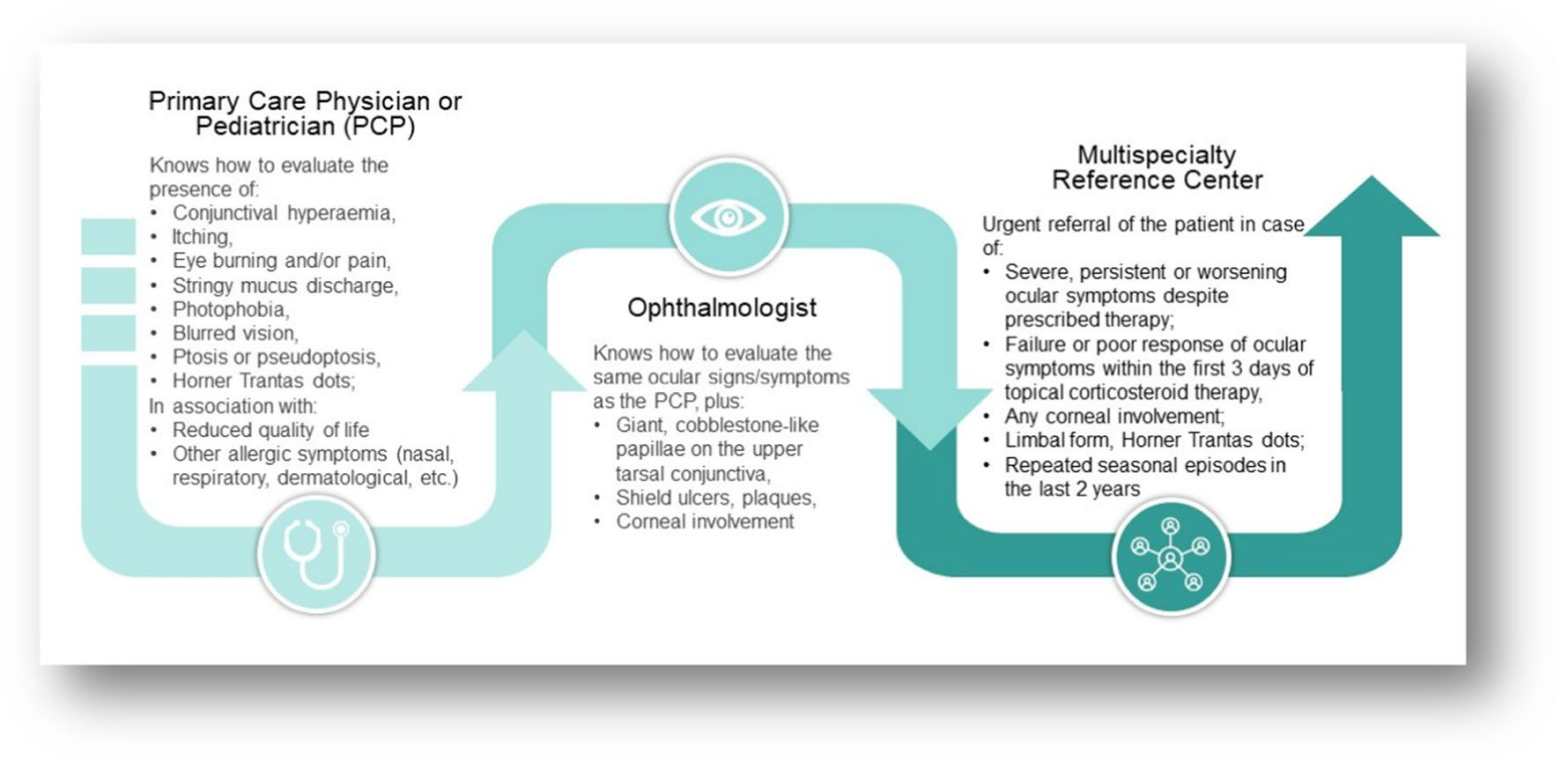
Relative roles of the primary care physician/pediatrician, ophthalmologist, and multispecialty reference center in the management of VKC

The acknowledgment of the current situation in VKC management, in both hospital and community settings, has prompted discussion among panel experts about the need for multispecialty reference centers. These centers are healthcare facilities that bring together experts from various medical specialties to provide coordinated care for patients with complex or specific health conditions, such as VKC. Indeed, reference centers should include not only pediatric ophthalmologists but also pediatric allergologists, given VKC’s classification as an ocular allergic disease, its associated comorbidities, and its predominant occurrence in pediatric patients [8–11]. Other specialists, such as immunologists and dermatologists, can also play important roles in the comprehensive management of patients with VKC [16]. However, the collaboration between ophthalmologists and pediatric allergologists has been deemed essential by the vast majority of experts. A collaborative approach within multispecialty reference centers may optimize resources, reduce costs, and enhance nationwide patient monitoring, minimizing the need for long-distance travel (i.e., medical tourism) during acute phases. In line with the current literature, the panelists also agree that nationally coordinated multispecialty reference centers should disseminate VKC knowledge locally, aiding primary care physicians/pediatrician to manage milder cases [16]. An enhanced understanding of the disease among general clinicians could streamline the referral process, thereby reserving specialized centers for patients with moderate to severe forms (Fig. 1).

In the United States (US), VKC is recognized by the National Organization for Rare Disorders (NORD). Obtaining VKC’s recognition as a rare disease in Europe and Italy as well would facilitate the creation of multispecialty reference centers, enhance medical professional training, alleviate patient and family burden, and ultimately optimize resource allocation [19].

For the initial diagnosis of VKC and for the diagnosis of subsequent flare-ups, it is advisable that the patient is not on corticosteroid therapy and has not received it for at least 7 days. However, sudden flare-ups may necessitate urgent outpatient evaluation, which is seldom available in reference centers. This situation poses a risk that inexperienced doctors might prescribe corticosteroid therapy before a definitive VKC diagnosis is made. Even when the patient is undergoing corticosteroid therapy, diagnosis is still possible, particularly in severe cases. Ophthalmological pictures taken during the acute phase may aid in diagnosing patients receiving corticosteroids.

The use of ocular cytology for VKC diagnosis is subject to ongoing debate. Children with active VKC who have not yet started therapy show a higher ocular expression of epithelial and inflammatory cells (neutrophils, mast cells, eosinophils, and lymphocytes) compared to those undergoing treatment. The cytological modifications of the ocular surface, as identified through conjunctival cytology, demonstrate a direct correlation with age of onset, duration, and severity of the disease [9, 24, 25]. However, some panelists highlighted challenges in routine outpatient settings, citing patient discomfort, time, costs, and the need for specialized personnel training as potential issues to consider. Others argued that a thorough patient history and clinical examination often suffice for a correct diagnosis, also pointing out that conjunctival cytological alterations are not specific to VKC [25, 26]. Therefore, the panel agreed on the use of ocular cytology only in research settings for the time being. Furter studies are needed to better explore ocular cytology’s role in clinical practice [27].

Once a diagnosis is made, severity should be defined. The most commonly used grading system for determining VKC severity is Italian, developed by a panel member (S.B.) and currently used internationally [24] (Table 3). The panel endorsed the use of this grading system, advocating for its national adoption after collegial reevaluation in light of the latest scientific evidence. The use of a common grading system will help standardize the diagnosis and, subsequently, treatment of VKC across the country. Indeed, a correct grading guides therapeutic approaches.

**Table 3:** VKC grading (modified from Bonini et al. [24])

Grade	Symptoms	Conjunctival hyperemia	Conjunctival secretion	Papillary reaction	Trantas dots	Corneal involvement
0 (quiescent)	Absent	Absent/mild	Absent	Mild to moderate	Absent	Absent
1 (mild intermittent)	Mild, occasional	Mild	Absent/mild	Mild to moderate	Absent	Absent
2A (moderate intermittent)	Mild to moderate, intermittent	Mild	Mild	Mild to severe	Absent	Absent
2B (moderate persistent)	Mild to moderate, persistent	Mild to moderate	Mild to moderate	Mild to severe	Absent	Superficial punctate keratitis
3 (severe)	Moderate to severe, persistent	Moderate to severe	Moderate to severe	Moderate to severe with injection and swelling	Few	Superficial punctate keratitis
4 (very severe)	Severe and persistent	Moderate to severe	Severe	Moderate to severe with injection and swelling	Numerous	Corneal erosions or ulcerations
5 (evolution)	Absent/mild, occasional	Absent/mild	Absent	Mild to severe fibrosis	Absent	Absent

**Fig. 2:**
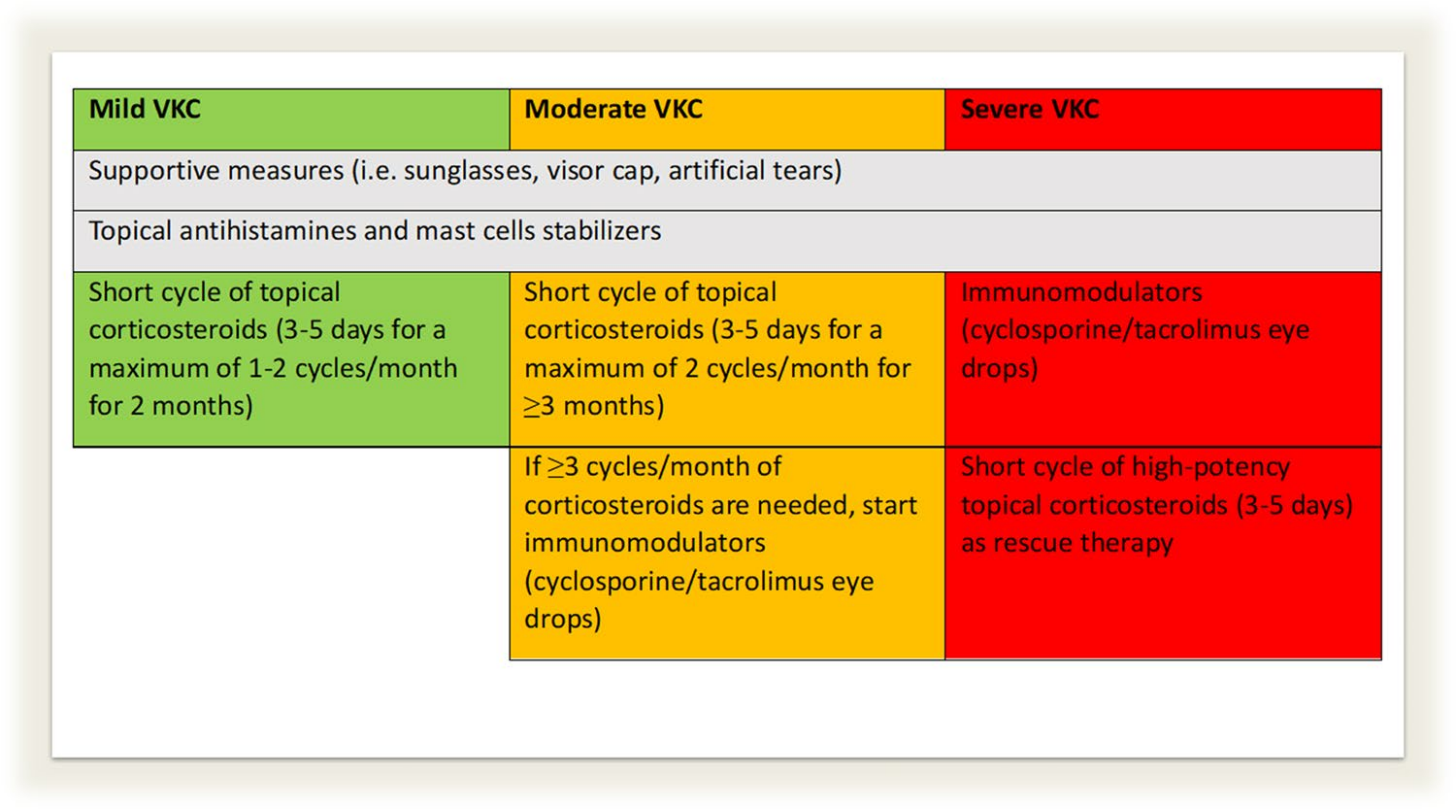
Therapeutic management of VKC as discussed and agreed upon by the expert panel members

Mild VKC typically responds to supportive measures (sunglasses, visor cap, artificial tears) and antihistamines and mast cell stabilizers, while moderate to severe cases may require corticosteroids or immunomodulators [5, 12, 19, 20] (Fig. 2). Such therapies should be prescribed by a specialist, particularly in newly diagnosed cases of VKC or in cases of uncertain diagnosis. In patients already diagnosed with VKC, antihistamine therapy is a first-line therapy that can be prescribed by general clinicians as per the 2023 European ophthalmological consensus [15]. For mild VKC cases resistant to first-line therapies, short corticosteroid courses have been proposed (3–5 days [11] up to twice a month and for a maximum of 2 consecutive months per year). In such scenarios, once the diagnosis has been established, general ophthalmologists can temporarily manage patients using topical corticosteroids, following the guidance of expert ophthalmologists. This strategy may help alleviate the burden on reference centers, particularly during peak periods of symptoms [28–33].

The use of topical corticosteroids requires an adequate understanding of the characteristics of the different molecules, which should be used according to the severity of VKC. Frequent ophthalmologic monitoring is essential to minimize the risks of iatrogenic ocular complications [28–33].

The prescription of low-penetration corticosteroids (hydrocortisone, clobetasone, budesonide, fluorometholone, loteprednol, difluprednate, and rimexolone) for short cycles is the preferred option in the mildest forms, with referrals to reference centers recommended if the disease persists or intensifies (> 2 months and/or > 2 courses of corticosteroids per month). The use of high-potency corticosteroids (e.g., dexamethasone) for short cycles without de-escalation is recommended in moderate cases, consistent with the European consensus [15], as they are considered more effective and have a lower risk of increasing intraocular pressure and inducing steroid dependence than low-potency corticosteroids (hydrocortisone, loteprednol).

In cases of prolonged corticosteroid therapies, immunomodulators (i.e., cyclosporine and tacrolimus) are needed as corticosteroid-sparing agents [5, 12, 19, 20, 34–36]. To date, cyclosporine for VKC is produced as eye drops at a concentration of 0.1% [35], while tacrolimus and other concentrations of cyclosporine are available only as galenic preparations. Exclusively in Japan, tacrolimus eye drops are produced at a 0.1% concentration (Talymus 0.1% Senju) [37]. In severe forms, at the beginning of immunomodulator therapy, the expert consensus favors the use of high-potency and high-penetration corticosteroids, given their ability to surpass the epithelial barrier, enabling a more rapid anti-inflammatory response and minimizing the risk of side effects associated with the prolonged use of corticosteroids, often required with milder formulations. Once the disease has been controlled with immunomodulators, there is no agreement among experts on rescue therapy cycles with corticosteroids, as some prefer to use high-potency high-penetration corticosteroids for short 3-day courses [35], while others prefer to use low-penetration corticosteroids, arguing that this reduces the risk of side effects. The efficacy of new biological drugs in VKC has not yet been evaluated, although their broad indications in chronic diseases suggest potential effectiveness in VKC patients.

## Strengths and limitations

To the best of our knowledge, this is the first consensus on VKC diagnosis and treatment to include both pediatricians and ophthalmologists [12, 15–18]. The present consensus relies on expert opinions, drawing on the most recent reviews and metanalyses [5, 7, 9, 11, 12, 15–17, 38]. By uniting experts from different fields (i.e., pediatric allergology and ophthalmology), our collaborative effort resulted in a unified and comprehensive guide for general clinicians caring for VKC patients. However, the fact that all panelists are Italian introduces a specificity to the Italian context, thereby limiting applicability on a global scale. Nonetheless, the localized nature proves valuable in standardizing approaches within the country. Future efforts may be directed toward the establishment of an international multidisciplinary consensus.

## Conclusions

By integrating insights from national ophthalmology and pediatric allergology experts, the present multidisciplinary consensus proposes a standardized approach to the diagnosis and treatment of VKC. Although the severity of VKC varies widely depending on many factors, general pediatricians and ophthalmologists can be trained to ensure prompt care and appropriate referrals. Disseminating knowledge based on the indications of reference centers may ease the management of mild VKC cases in local settings.

## Supplementary Information

Below is the link to the electronic supplementary material.

Supplementary file1 (DOCX 55 KB)

## Data Availability

No datasets were generated or analysed during the current study.

## Abbreviations

PCP: Primary care physician/pediatrician
SIAIP: Italian Society of Pediatric Allergy and Immunology
SIOPS: Italian Society of Pediatric Ophthalmology and Strabismus
VKC: Vernal keratoconjunctivitis

## References

[1] Bonini S, Bonini S, Lambiase A et al (2000) Vernal keratoconjunctivitis revisited: a case series of 195 patients with long-term followup. Ophthalmology 107:1157–1163. 10.1016/s0161-6420(00)00092-0 10857837 10.1016/s0161-6420(00)00092-0

[2] Bremond-Gignac D, Donadieu J, Leonardi A et al (2008) Prevalence of vernal keratoconjunctivitis: a rare disease? Br J Ophthalmol 92:1097–1102. 10.1136/bjo.2007.117812 18356259 10.1136/bjo.2007.117812

[3] Montan PG, Ekström K, Hedlin G et al (1999) Vernal keratoconjunctivitis in a Stockholm ophthalmic centre–epidemiological, functional, and immunologic investigations. Acta Ophthalmol Scand 77:559–563. 10.1034/j.1600-0420.1999.770516.x 10551301 10.1034/j.1600-0420.1999.770516.x

[4] Leonardi A, Busca F, Motterle L et al (2006) Case series of 406 vernal keratoconjunctivitis patients: a demographic and epidemiological study. Acta Ophthalmol Scand 84:406–410. 10.1111/j.1600-0420.2005.00622.x 16704708 10.1111/j.1600-0420.2005.00622.x

[5] Kumar S (2009) Vernal keratoconjunctivitis: a major review. Acta Ophthalmol 87:133–147. 10.1111/j.1755-3768.2008.01347.x 18786127 10.1111/j.1755-3768.2008.01347.x

[6] Zicari AM, Nebbioso M, Lollobrigida V et al (2013) Vernal keratoconjunctivitis: atopy and autoimmunity. Eur Rev Med Pharmacol Sci 17:1419–1423 23740459

[7] Sacchetti M, Plateroti R, Bruscolini A et al (2021) Understanding vernal keratoconjunctivitis: beyond allergic mechanisms. Life (Basel) 11:1012. 10.3390/life11101012 34685384 10.3390/life11101012 PMC8541022

[8] Fauquert J-L (2019) Diagnosing and managing allergic conjunctivitis in childhood: The allergist’s perspective. Pediatr Allergy Immunol 30:405–414. 10.1111/pai.13035 30742722 10.1111/pai.13035

[9] Bruschi G, Ghiglioni DG, Cozzi L et al (2023) Vernal keratoconjunctivitis: a systematic review. Clin Rev Allergy Immunol 65:277–329. 10.1007/s12016-023-08970-4 37658939 10.1007/s12016-023-08970-4 PMC10567967

[10] Zicari AM, Capata G, Nebbioso M et al (2019) Vernal keratoconjunctivitis: an update focused on clinical grading system. Ital J Pediatr 45:64. 10.1186/s13052-019-0656-4 31113464 10.1186/s13052-019-0656-4 PMC6528205

[11] Leonardi A (2013) Management of vernal keratoconjunctivitis. Ophthalmol Ther 2:73–88. 10.1007/s40123-013-0019-y 25135808 10.1007/s40123-013-0019-y PMC4108143

[12] Mehta JS, Chen W-L, Cheng ACK et al (2022) Diagnosis, management, and treatment of vernal keratoconjunctivitis in asia: recommendations from the management of vernal keratoconjunctivitis in Asia Expert Working Group. Front Med (Lausanne) 9:882240. 10.3389/fmed.2022.882240 35979210 10.3389/fmed.2022.882240 PMC9376221

[13] Smedt SD, Nkurikiye J, Fonteyne Y et al (2011) Vernal keratoconjunctivitis in school children in Rwanda and its association with socio-economic status: a population-based survey. Am J Trop Med Hyg 85:711–717. 10.4269/ajtmh.2011.11-0291 21976577 10.4269/ajtmh.2011.11-0291 PMC3183782

[14] Ghauri A-J, Fisher K, Kenworthy A (2021) Understanding the journey of patients with vernal keratoconjunctivitis: a qualitative study of the impact on children and families. J Pediatr Ophthalmol Strabismus 58:298–303. 10.3928/01913913-20210319-01 34180284 10.3928/01913913-20210319-01

[15] Dahlmann-Noor A, Bonini S, Bremond-Gignac D et al (2023) Novel insights in the management of vernal keratoconjunctivitis (VKC): European Expert Consensus using a modified nominal group technique. Ophthalmol Ther 12:1207–1222. 10.1007/s40123-023-00665-5 36790673 10.1007/s40123-023-00665-5 PMC10011216

[16] Ghauri A-J, Biswas S, Manzouri B et al (2023) Management of vernal keratoconjunctivitis in children in the United Kingdom: a review of the literature and current best practice across six large United Kingdom centers. J Pediatr Ophthalmol Strabismus 60:6–17. 10.3928/01913913-20220328-01 35611818 10.3928/01913913-20220328-01

[17] Miyazaki D, Takamura E, Uchio E et al (2020) Japanese guidelines for allergic conjunctival diseases 2020. Allergol Int 69:346–355. 10.1016/j.alit.2020.03.005 33211650 10.1016/j.alit.2020.03.005

[18] Takamura E, Uchio E, Ebihara N et al (2017) Japanese guidelines for allergic conjunctival diseases 2017. Allergol Int 66:220–229. 10.1016/j.alit.2016.12.004 28209324 10.1016/j.alit.2016.12.004

[19] Ghiglioni DG, Zicari AM, Parisi GF et al (2021) Vernal keratoconjunctivitis: an update. Eur J Ophthalmol 31:2828–2842. 10.1177/11206721211022153 34058899 10.1177/11206721211022153

[20] Esposito S, Fior G, Mori A et al (2016) An update on the therapeutic approach to vernal keratoconjunctivitis. Paediatr Drugs 18:347–355. 10.1007/s40272-016-0185-1 27461427 10.1007/s40272-016-0185-1

[21] Leonardi A, Mori F, Ghiglioni DG (2023) A survey-based study on diagnosis and management of vernal keratoconjunctivitis. Pediatr Allergy Immunol 34:e13962. 10.1111/pai.13962 37232283 10.1111/pai.13962

[22] Gattrell WT, Logullo P, van Zuuren EJ et al (2024) ACCORD (ACcurate COnsensus Reporting Document): a reporting guideline for consensus methods in biomedicine developed via a modified Delphi. PLoS Med 21:e1004326. 10.1371/journal.pmed.1004326 38261576 10.1371/journal.pmed.1004326 PMC10805282

[23] Diamond IR, Grant RC, Feldman BM et al (2014) Defining consensus: a systematic review recommends methodologic criteria for reporting of Delphi studies. J Clin Epidemiol 67:401–409. 10.1016/j.jclinepi.2013.12.002 24581294 10.1016/j.jclinepi.2013.12.002

[24] Bonini S, Sacchetti M, Mantelli F, Lambiase A (2007) Clinical grading of vernal keratoconjunctivitis. Curr Opin Allergy Clin Immunol 7:436–441. 10.1097/ACI.0b013e3282efb726 17873585 10.1097/ACI.0b013e3282efb726

[25] Thatte S, Varma A, Kapadia S et al (2021) Impression cytology screening for ocular surface changes in various forms of vernal keratoconjunctivitis in subtropical central zone in India. Nepal J Ophthalmol 13:105–117. 10.3126/nepjoph.v13i2.32838 35996777 10.3126/nepjoph.v13i2.32838

[26] Bruschi G, Ghiglioni DG, Osnaghi S et al (2020) Role of ocular cytology in vernal keratoconjunctivitis. Immun Inflamm Dis 8:3–7. 10.1002/iid3.278 31804769 10.1002/iid3.278 PMC7016839

[27] Heffler E, Landi M, Caruso C et al (2018) Nasal cytology: methodology with application to clinical practice and research. Clin Exp Allergy 48:1092–1106. 10.1111/cea.13207 29904978 10.1111/cea.13207

[28] St Clair Roberts D (1986) Steroids, the eye, and general practitioners. Br Med J (Clin Res Ed) 292:1414–1415. 10.1136/bmj.292.6533.1414 10.1136/bmj.292.6533.1414 PMC1340426 3087452

[29] van Rensburg EJ, Meyer D (2013) Astute and safe use of topical ocular corticosteroids in general practice: practical guidelines. Contin Med Educ 31:396–398

[30] Lam DSC, Fan DSP, Ng JSK et al (2005) Ocular hypertensive and anti-inflammatory responses to different dosages of topical dexamethasone in children: a randomized trial. Clin Exp Ophthalmol 33:252–258. 10.1111/j.1442-9071.2005.01022.x 15932528 10.1111/j.1442-9071.2005.01022.x

[31] Fernando P, Marziali E, Chlubek M et al (2021) Pulsed oral corticosteroids for the treatment of vernal and atopic keratoconjunctivitis: a management plan. Eye (Lond) 35:1277–1278. 10.1038/s41433-020-1062-2 32612172 10.1038/s41433-020-1062-2 PMC8115223

[32] Lavin MJ, Rose GE (1986) Use of steroid eye drops in general practice. Br Med J (Clin Res Ed) 292:1448–1450. 10.1136/bmj.292.6533.1448 3087466 10.1136/bmj.292.6533.1448 PMC1340444

[33] Jones R, Rhee DJ (2006) Corticosteroid-induced ocular hypertension and glaucoma: a brief review and update of the literature. Curr Opin Ophthalmol 17:163–167. 10.1097/01.icu.0000193079.55240.18 16552251 10.1097/01.icu.0000193079.55240.18

[34] Brindisi G, Cinicola B, Anania C et al (2021) Vernal keratoconjunctivitis: state of art and update on treatment. Acta Biomed 92:e2021517. 10.23750/abm.v92iS7.12419 34842588 10.23750/abm.v92iS7.12419 PMC9431888

[35] Leonardi A, Doan S, Amrane M et al (2019) A randomized, controlled trial of cyclosporine a cationic emulsion in pediatric vernal keratoconjunctivitis: the VEKTIS study. Ophthalmology 126:671–681. 10.1016/j.ophtha.2018.12.027 30593775 10.1016/j.ophtha.2018.12.027

[36] Leonardi A, Silva D, Perez Formigo D et al (2019) Management of ocular allergy. Allergy 74:1611–1630. 10.1111/all.13786 30887530 10.1111/all.13786

[37] Hirota A, Shoji J, Inada N et al (2022) Evaluation of clinical efficacy and safety of prolonged treatment of vernal and atopic keratoconjunctivitis using topical tacrolimus. Cornea 41:23–30. 10.1097/ICO.0000000000002692 34870621 10.1097/ICO.0000000000002692 PMC8647698

[38] Gokhale NS (2016) Systematic approach to managing vernal keratoconjunctivitis in clinical practice: severity grading system and a treatment algorithm. Indian J Ophthalmol 64:145–148. 10.4103/0301-4738.179727 27050351 10.4103/0301-4738.179727 PMC4850811

