# Supplementary material for: Seeing eye to eye: a modified Delphi method-based multidisciplinary expert consensus on the diagnosis and treatment of vernal keratoconjunctivitis
Source: Eur J Pediatr. 2024 Sep 26;183(11):5053–61. doi: 10.1007/s00431-024-05776-0 (PMC11473654; doi:10.1007/s00431-024-05776-0)
Supplement: Supplementary file 1 — Supplementary file1 (DOCX 55 KB) [file 431_2024_5776_MOESM1_ESM.docx]

**Supplementary Table 1. Overview of the 3 rounds in the consensus development process leading to the formulation of the final 10 statements.**

| **Rationale** | **First round** | **Degree of agreement** | **Second round** | **Degree of agreement** | **Third round** | **Degree of agreement** |
| --- | --- | --- | --- | --- | --- | --- |
| The absence of definitive studies on the optimal diagnostic and management strategies for Vernal Keratoconjunctivitis (VKC) poses a challenge[8, 10–12, 20, 24]. Despite VKC being primarily an ophthalmological condition[11], its occurrence in childhood and its correlation with other conditions, particularly allergic-immunological and dermatological disorders, highlights the need for a collaborative approach[8]. Particularly in Italy, which can count on a pediatric national health system, the involvement of pediatricians, allergologists, immunologists, dermatologists and ophthalmologists is crucial to achieve accurate diagnosis and effective follow-up for VKC patients[20]. | For the diagnosis and follow-up of VKC it is recommended to conduct ophthalmological and allergological examinations. In severe forms, seeking advice from other expert medical specialists, possibly in multispecialty reference centers, is advisable. | 88% |  |  |  |  |
| The progression of VKC is influenced by seasonal, environmental and atmospheric factors, introducing multiple variables in disease assessment. Comparative studies on the exclusive involvement of expert ophthalmologists versus multispecialty reference centers in the diagnosis and management of VKC are currently lacking. However, existing research emphasizes that training pediatricians, allergologists and ophthalmologists facilitates early diagnosis and improves the overall effectiveness of VKC management[8, 11]. | Multispecialty reference centers should develop written instructions and information for patients, their families and primary care physicians/pediatricians regarding the in-home management of mild forms of VKC. | 88% |  |  |  |  |
| Research has yet to provide insights into the optimal clinical conditions for diagnosing VKC or its recurrence[5, 19]. However, for an accurate staging of the severity of the disease, the patient should present an active disease, hence without ongoing corticosteroid therapy. | The initial clinical diagnosis and assessment of VKC exacerbations should be promptly conducted in patients exhibiting active disease who have not received topical or systemic corticosteroid therapy for a minimum of 7 days. | 82% |  |  |  |  |
| The diagnosis of VKC and its flare-ups relies on clinical history, symptoms, signs and environmental/personal factors, as no biochemical markers are currently available in routine practice[5, 10, 11, 19, 20]. The absence of standardized criteria is reflected in the diverse severity classifications and diagnostic-therapeutic protocols employed by different reference centers and experts, highlighting the need for a more universally applicable framework[24]. | The severity of the disease should be assessed through a score shared by reference centers including signs, symptoms and the presence of aggravating factors. | 94% |  |  |  |  |
| In children with active VKC who have not yet started therapy, there is a higher expression of epithelial and inflammatory cells (neutrophils, mast cells, eosinophils, and lymphocytes) compared to children undergoing treatment. This suggests that topical therapy can influence the cytological composition of the conjunctiva in VKC, aligning with observations in other severe and chronically relapsing ocular surface pathologies. The cytological modifications of the ocular surface, as identified through conjunctival cytology, show a direct correlation with age of onset, duration and severity of the disease[25, 26]. Conjunctival cytology may provide complementary diagnostic information that could guide therapeutic decisions. | Ocular cytology should be performed at the time of diagnosis and in case of recrudescence as well as every 3 months in severe forms to monitor the disease during therapy. | 53% | Conjunctival cytology should be performed at diagnosis especially in severe and/or dubious forms of VKC and in the context of clinical trials. Serial conjunctival cytologies should be performed over time, as well as in the context of clinical studies and/or in the case of unexpected evolution or recurrence to identify overlapping and intercurrent causes (e.g., infections). Conjunctival cytology should be performed through minimally traumatic methods and by expert personnel, to make the procedure more acceptable even in the first years of life. | 59% | Conjunctival cytology currently plays a limited diagnostic role in everyday clinical practice and is often reserved to clinical trials. Its use is further constrained by the necessity for skilled personnel, aiming to minimize both the time and costs of implementation, as well as potential stress for the patient. In rare instances where the clinical differential diagnosis poses particular challenges, conjunctival cytology may offer supplementary insights contributing to the overall diagnostic process. | 94% |
| Studies have not yet provided conclusive evidence regarding the extent to which supportive measures (e.g., sunglasses, visor caps, artificial tears) and therapy with topical antihistamines and mast cell stabilizers can alleviate symptoms and reduce signs in mild forms or early stages of VKC, thus delaying the need for other medications (steroids and immunomodulators). However, a growing body of research suggests that appropriate use of supportive therapies can effectively mitigate symptoms, potentially reducing the reliance on more potent medications (steroids and immunomodulators), thereby aiding in the management of mild VKC forms[5, 11, 12]. | In mild forms of VKC and during flare-ups, it is essential to instruct caregivers on the implementation of supportive measures (eye wash, sunglasses, visor cap, artificial tears), along with the use of topical antihistamines and mast cell stabilizers, before considering referral to a reference center. | 71% | Supportive measures (particularly sunglasses, visor cap, artificial tears), and antihistamines and topical mast cell stabilizers in case of hitching and/or known allergy should be recommended in the in-home management of all forms of VKC. In milder forms, the proper use of these aids can effectively control the disease, while in moderate forms it can delay the need for corticosteroids and immunomodulators. | 88% |  |  |
| When supportive therapies and antihistamines prove ineffective, several studies suggest considering topical corticosteroid therapy in various modalities. In VKC, as in other ocular pathologies, prolonged local steroid therapy is not recommended, due to potential side effects. Therapy with immunomodulators is generally recommended when VKC is not controlled with topical corticosteroids or is controlled only with prolonged use of topical corticosteroids. In particularly severe forms, therapy with immunomodulators is started immediately and corticosteroids are used as rescue therapy[11, 36]. | In individuals with VKC, corticosteroids should be administered at home under the guidance of the general practitioner, adhering to specialist recommendations outlined by reference centers. | 74% | In individuals with VKC, topical steroid therapy should be prescribed and monitored by the expert ophthalmologist and/or the multispecialty reference center. The general physician/pediatrician should be informed of the therapy to facilitate its correct administration and schedule ophthalmological visits based on clinical evolution and according to the indications of the multispecialty referencecenter. | 82% |  |  |
| There is a lack of comparative studies addressing the optimal use of topical corticosteroids for controlling VKC, across its mild, moderate, and severe forms. Only in a randomized controlled international study was a high-penetration corticosteroid employed as rescue therapy, integrated into the course of cyclosporine eye drop therapy. Prior to initiating immunomodulator therapy, there are reports of surface corticosteroids being used in decreasing cycles of 15-20 days, or high-penetration and high-potency corticosteroids being used in cycles of 3-5 days[35]. | In the acute phase and in severe forms, short-term steroid cycles (3 days) are preferable for VKC control compared to cycles of 15-21 days. | 71% | In mild forms with periods of recrudescence of less than 3 months, steroid eye drops should be used for decreasing cycles of 15-20 days. In moderate forms, steroid eye drops should be used for short cycles of 3-5 days, repeatable up to a maximum of three times in a month, before starting the patient on immunomodulators. In severe forms treated with immunomodulators, steroid eye drops should be used for short cycles of 3-5 days that can be repeated as rescue therapy. | 70% | In mild forms with periods of recrudescence of less than 3 months, steroids should not be used or should be used in short cycles of 3-5 days 1-2 times a month. In moderate forms and with periods of recrudescence of 3 or more months, steroids should be used in short cycles of 3-5 days. If 3 or more steroid cycles are needed in a month to control the disease, they should be replaced with immunomodulators. In severe forms requiring treatment with immunomodulators, steroids should be promptly used as supportive anti-inflammatory therapy for a brief duration at first, followed by short and repeatable 3-5 day cycles as rescue therapy, if needed. | 88% |
| There is a lack of comparative studies on the use of corticosteroids of varying potency and ocular penetration for VKC disease control. While surface corticosteroids might be preferable, in routine practice steroids with low and high penetration are often used interchangeably. In an international study[35], higher potency corticosteroids are favored as rescue therapy during immunomodulator treatment (cyclosporine eye drops). Nevertheless, there are instances in the literature where corticosteroids of varying potencies and penetrations have been utilized before initiating immunomodulator therapy. | High-penetration corticosteroids are to be preferred over low-penetration corticosteroids in severe VKC. | 65% | In severe cases of VKC, higher potency steroids are preferable to lower potency steroids. Steroid eye drops with lower ocular penetration and fewer eye-irritating preservatives/excipients should also be preferred. | 82% |  |  |
| In 8-15% of cases cyclosporine eye drop therapy may not effectively control VKC[19]. The definition of lack of control varies among reference centers due to different classifications and the use of varied supportive steroid therapy in different centers. Prolonged or repeated use of corticosteroids during cyclosporine eye drop therapy is an indication to replace the current therapy with tacrolimus galenic eye drops (0.1%). | Tacrolimus 0.1% galenic eye drops are an alternative in case of ineffective therapy with cyclosporine eye drops. | 82% |  |  |  |  |
